# Complete Genome Sequence of a Novel Bioflocculant-Producing Strain, *Microbacterium paludicola* CC3

**DOI:** 10.1128/genomeA.01008-17

**Published:** 2017-09-21

**Authors:** Weijie Liu, Cong Liu, Di Sun

**Affiliations:** School of Life Science, The Key Laboratory of Biotechnology for Medicinal Plants of Jiangsu Province, Jiangsu Normal University, Xuzhou, Jiangsu Province, China

## Abstract

*Microbacterium paludicola* CC3 exhibits the capability to produce polysaccharide bioflocculants. Here, we report the whole-genome sequence of *M. paludicola* CC3, which may be helpful in understanding the genetic basis of the biosynthesis of polysaccharide bioflocculants as well as in promoting its production and application in industrial fields.

## GENOME ANNOUNCEMENT

Bioflocculants are mainly extracellular polymeric substances secreted by microorganisms (1) and are widely applied in microalgae harvest (2) and wastewater treatment (3), due to their harmless and biodegrading properties (4). The genome of several strains that can produce bioflocculants have been sequenced, including those of *Paenibacillus shenyangensis*, *Agrobacterium tumefaciens* F2, and *Paenibacillus wulumuqiensis* (5–7). However, the genomic data of bioflocculant-producing strains are still rare, which limits the identification of key enzymes and metabolic pathways that are involved in the biosynthesis of bioflocculants.

In this study, a novel bioflocculant-producing strain, *Microbacterium paludicola* CC3, was sequenced with the Pacific Biosciences (PacBio) RSII platform using P6-C4 chemistry. The resulting sequencing reads with 320.4-fold coverage were then *de novo* assembled using Hierarchical Genome Assembly Process (HGAP) (8, 9). Gene prediction was performed against the assembled CC3 genome with GeneMarkS (10). Functional characterization of predicted genes was based on a BLASTP search against GenBank’s nonredundant (NR) protein database, the database of the Clusters of Orthologous Groups of proteins (COG) (http://www.ncbi.nlm.nih.gov/COG/), and the Gene Ontology (GO) Consortium (http://www.geneontology.org/). The metabolic pathways were predicted using the KEGG Automatic Annotation Server (KAAS) (http://www.genome.jp/tools/kaas/). rRNAs and tRNAs were identified using Barrnap 0.4.2 (http://www.vicbioinformatics.com/software.barrnap.shtml) and tRNAscan-SE version 2.0 (http://lowelab.ucsc.edu/tRNAscan-SE/), respectively. The clustered regularly interspaced short palindromic repeat (CRISPR) elements were detected using PILER-CR (11).

One gapless circular contig was assembled, which corresponded to the chromosome of *M. paludicola* CC3. No plasmid sequences were detected. The chromosome was composed of 3,410,829 bp, with an average G+C content of 70.10%, which comprised 3,390 predicted genes, of which 3,209 were protein coding genes (CDSs), 32 were tRNA genes, 146 were rRNA genes, and 3 were microRNA genes. Pseudogenes and prophage genes were not identified, whereas 14 CRISPR candidates were detected in the genome of strain CC3. A series of genes encoding polysaccharide biosynthesis/modification proteins, such as mannose-1-phosphate guanylyltransferase (12), glucose-1-phosphate thymidylyltransferase (13), dolichol-phosphate mannosyltransferase (14), dTDP-4-dehydrorhamnose reductase (15), and genes involved in polysaccharide ABC-type transporters were detected, which may function in the biosynthesis of polysaccharide bioflocculant and transportation across membranes and cell walls (16, 17). In previous studies, the biomass wastes were directly used as carbon source of lignocellulose-degrading strains to produce various value-added products at a low cost (2, 18). Therefore, we are interested in the genes encoding the lignocellulose-degrading enzymes. Genes encoding xylanase, cellulose, and amylase were identified, thereby suggesting that strain CC3 can directly convert biomass waste into polysaccharide bioflocculants.

### Accession number(s).

The sequence data for the genome of *M. paludicola* CC3 have been deposited to GenBank under the accession number CP018134; the version described in this paper is the first version. Strain CC3 has been deposited at the China General Microbiological Culture Collection Center (CGMCC 1.15930).
